# Microchamber Device for Detection of Transporter Activity of Adherent Cells

**DOI:** 10.3389/fbioe.2015.00032

**Published:** 2015-03-18

**Authors:** Mamiko Tsugane, Etsuko Uejima, Hiroaki Suzuki

**Affiliations:** ^1^Department of Precision Mechanics, Faculty of Science and Engineering, Chuo University, Tokyo, Japan; ^2^Department of Clinical Pharmacy Research and Education, Graduate School of Pharmaceutical Sciences, Osaka University, Osaka, Japan

**Keywords:** microchamber, transporter, transport assay, anti-cancer drug, adherent cell

## Abstract

We present a method to detect the transporter activity of intact adherent cells using a microchamber device. When adherent cells are seeded onto the poly-di-methyl siloxane substrate having microchambers with openings smaller than the size of a cell, the cells form a confluent layer that covers the microchambers, creating minute, confined spaces. As substances exported across the cell membrane accumulate, transporter activity can be detected by observing the fluorescence intensity increase in the microchamber. We tested the microchamber device with HeLa cells over-expressing MDR1, an ATP-binding cassette transporter, and succeeded in detecting the transport of fluorescence-conjugated paclitaxel, the anti-cancer drug, at the single-cell level.

## Introduction

Cells take up and release various substances across the cellular membrane. The underlying mechanisms include passive diffusion, substance-specific passive or active transport via membrane proteins, and endo- and exocytosis through vesicles. Through these mechanisms a variety of substances are transported, such as fat-soluble to aqueous-soluble substances and small molecules to large substances. Because membrane transport plays a key role in various physiological activities, the development of methods to measure the transport of such substances is expected to have a wide range of applications, from basic biological sciences to clinical applications.

Among membrane proteins that transport physiologically active substances (e.g., glucose, amino acids) between the exterior and interior of cells are the transporter proteins. Since transporters are also involved in the efflux of drugs, they have an impact on pharmacokinetics. Above all, the efflux of anti-cancer drugs by ATP-binding cassette (ABC) transporters expressed on cancer cells is of particular importance (Leonard et al., 2003; Szakacs et al., 2006; Huang, 2007; Mellor and Callaghan, 2008). This drug transporter induces multi-drug resistance, which shows resistance against anti-cancer drugs with many different structures or mechanisms of action in cancer cells. Thus, ABC transport is considered to be the major factor in cases where the drug is not effective. ABC transporters export substances by using energy derived from ATP hydrolysis (Klein et al., 1999). Among various subfamilies, *MDR1/ABCB1*, *MRP1/ABCC1*, and *BCRP/ABCG2* are primarily involved in anti-cancer resistance (Gottesman et al., 2002). A transporter encoded in the *MDR1/ABCB1* gene is called P-glycoprotein, which is expressed in normal cells such as those in the kidneys and adrenal glands, and plays a role in biological defense by exporting toxic substances. When P-glycoprotein is over-expressed in cancer cells, anti-cancer drugs will be exported to the outside of the cells and the intracellular accumulation of the drug decreases, leading to the acquisition of resistance by the cancer cells. MDR1 has a wide range of substrate specificities and is involved in resistance against diverse chemical structures, including anthracyclines, vinca alkaloids, and taxanes (Thomas and Coley, 2003).

Several analytical methods are available to quantify the transport activity of either transporter-expressing cells or vesicles with reconstituted transporters (e.g., scintillation counter, fluorescent plate reader, flow cytometry) (Aszalos, 2007; Giacomini et al., 2010). However, these methods are endpoint assays and transport dynamics cannot be resolved. To directly quantify the transport of substrates across the cellular membrane, the transwell device is widely used. In this device, a confluent monolayer of cells with tight occluding junctions (e.g., Caco-2, MDCK, LLC-PK1) is prepared over a membrane with micrometric holes. As the transporters are expressed only on the apical side, directional transport of substrates across the cell layer can be measured. These methods are used widely to screen drugs against certain transporters. However, these methods only provide the average behavior of a cellular population. It has been widely recognized that, among cells derived from the same tissue, there are wide genetic and functional varieties (Michor and Polyak, 2010; Visvader, 2011; Renovanz and Kim, 2014). The heterogeneity in cancer cells, especially, may affect drug efficacy and reduce the quality of treatment. Therefore, a simple device that can measure the transport activity of cells at the single-cell level would be beneficial to analyze clinical samples. Moreover, such a device could be expected to reduce the amount of cells that need to be analyzed, alleviating the burden on patients.

With the advance of micro total analysis system technology (μTAS), various microdevices for singe-cell and single-molecule analysis are becoming available, not only for fundamental biology but also for clinical diagnostics (Sims and Allbritton, 2007; Kovarik et al., 2012; Culbertson et al., 2014). Several devices that measure the activity of transporters have been reported so far. Unlike ion channels, whose activity can be measured electrically, substrates of transporters are various and often have no charge. For this reason, detection of metabolites exported via transporters is principally dependent on fluorescence, which is monitored using fluorescence microscopes. When transported substrates accumulate in a small, closed space, high concentrations can be achieved within a short period of time, leading to highly sensitive detection.

Measurement of the transport activity of non-adherent cells at a single-cell level can be realized by trapping cells within a microchamber or microfluidics device and directly observing the translocation of fluorescent substrate. Iino et al. (2012) reported a screening method for drug resistant bacteria: *Escherichia coli* was trapped together with fluorogenic substrates within a femtoliter chamber or droplet array and the localization of fluorescent substrates degraded by β-galactosidase was examined. In addition, Li et al. (2008, 2011) reported a method to monitor the efflux and accumulation of fluorescent anti-cancer drugs for a single cell from the leukemic (CEM) cell line, trapped by a retention structure on a microfluidic chip.

Moreover, the measurement of membrane transport at a single transporter molecule level has been attempted. Tschodrich-Rotter and Peters (1998) attached the membranes of erythrocytes onto an isoporous polycarbonate filter, and the passive transport of fluorescent protein B-phycoerythrin through pores created by streptolysin O was detected. Kiskin et al. (2003) used a similar method to measure passive transport of nuclear transport receptor NTF2 and its homolog NXT1 through pores in nuclear membranes of *Xenopus* oocytes using a microchamber (Kiskin et al., 2003; Peters, 2003). Furthermore, several groups have succeeded in detecting the activity of single transporters embedded in artificial, reconstituted membranes that cover a microchamber (Hemmler et al., 2005; Barriga et al., 2014; Watanabe et al., 2014). In these pilot experiments, the performance of microdevices in detecting passive transport was primarily tested, due to the ease of experiment, although they expect the same strategy will enable the measurement of active transport.

**Figure 1:**
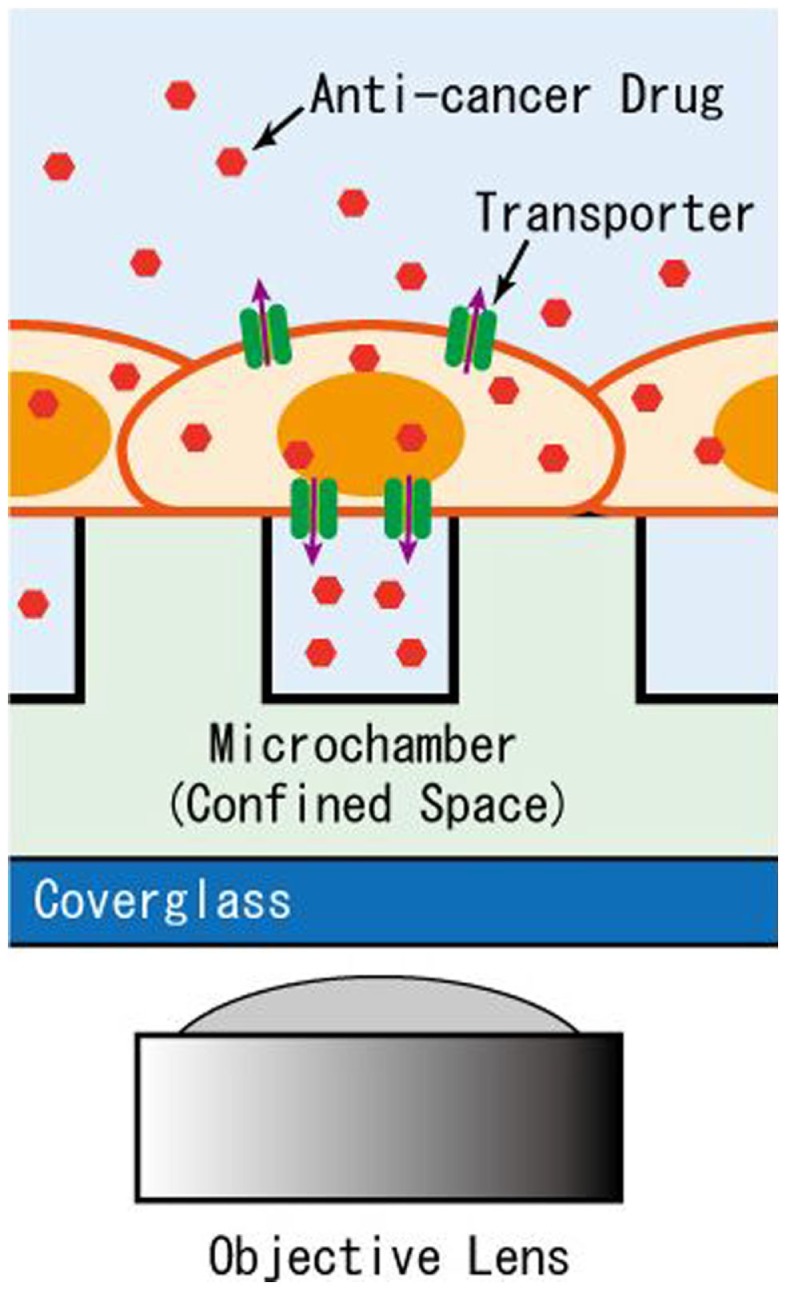
**Schematic of the microchamber system for detection of transporter activity expressed in adherent cells**. The efflux of anti-cancer drug accumulates in the microchamber sealed by the intact cells for detection by imaging.

In the pharmacotherapy of cancer, there is wide variation among individuals in treatment efficacy and the development of side effects from anti-cancer drugs, due to the acquisition of resistance by cancer cells as well as polymorphisms in patients. A clinical diagnostic device that detects the activity of transporters based on cells collected from cancer patient biopsies could help predict the efficacy and side effects of certain drugs, and contribute to the selection of appropriate anti-cancer treatments for individual patients. As seen in the above examples, transport of substances via membrane transporters can be detected rapidly and easily at the single-cell or single-molecule level using microdevices. As solid tumors account for most tumors that consist of adherent cells, a microdevice that measures transport activity of adherent cells under near-physiological conditions is of particular importance. Thus, in this study, we develop a microchamber device for the detection of transport activity, in which drugs, as substrates, are exported from cells and accumulate within a confined space of the microchamber directly covered by adherent, cultured cells (Figure 1). Here, cultured cells over-expressing MDR1 proteins were used to test the performance of the constructed microchamber device in measuring the transport activity of anti-cancer drugs.

## Materials and Methods

### Reagent

NaCl, KCl, CaCl_2_, MgCl_2_, NaOH, and dimethysulfoxide (DMSO) were purchased from Sigma-Aldrich (St. Louis, MO, USA). 4-(2-hydroxyethyl) piperazine-1-ethanesulfonicacid (HEPES) was purchased from DOJINDO (Kumamoto, Japan). Glucose was purchased from Wako Pure Chemicals Industries (Osaka, Japan).

### Cell culture

A human cervical carcinoma (HeLa) cell line was acquired from the Japanese Cancer Research Resources Bank (JCRB). Cells were cultured in Dulbecco’s modified Eagle’s medium (DMEM; Sigma-Aldrich) supplemented with 10% fetal bovine serum (FBS; Life Technologies Carlsbad, CA, USA) and 50 U/ml penicillin/streptomycin (P/S; Sigma-Aldrich) at 37°C in 5% CO_2_. For the microchamber experiment, cells cultured on dishes were removed using a 0.25% trypsin-EDTA solution (Sigma-Aldrich) and suspended in a basal salt solution (BSS) consisting of 130 mM NaCl, 5.4 mM KCl, 1.8 mM CaCl_2_, 0.8 mM MgCl_2_, 5.5 mM glucose, and 10 mM Hepes-NaOH (pH 7.3). The cell concentration was adjusted to 3.0 × 10^7^ cell/ml for seeding on the microchamber device. This cell concentration was optimized to achieve confluency at about 90 min of incubation after seeding.

### Transfection

HeLa cells were cultured on plastic Petri dishes containing media without P/S for 24 h. Cells at 60–80% confluency were transfected with a plasmid expressing the ABC transporter protein MDR1 using lipofectamine LTX (Life Technologies). A plasmid vector with green fluorescent protein (GFP)-tagged ORF clones of human ABC, sub-family B, member 1 (*MDR1/ABCB1*) was purchased from ORIGENE (Rockville, MD, USA). Cells were supplemented with a solution containing plasmid DNA, a transfection reagent, and Opti-MEM1 Reduced-Serum Medium (Life Technologies), and were cultured for 24–48 h.

### Fabrication and preparation of the microchamber device

**Figure 2:**
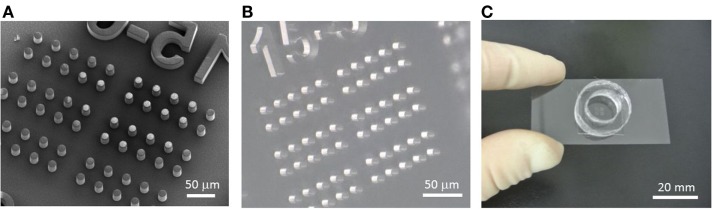
**Fabrication process of the microchamber device**. **(A)** SEM image of the SU-8 master mold formed on a silicon wafer. **(B)** PDMS microchamber device removed from the mold. **(C)** Microchamber device with the ring structure used to retain the buffer and reagents. This setup is mounted on the microscope stage for imaging.

A microchamber device made of poly-di-methyl siloxane (PDMS) was fabricated by the standard soft-lithography process. First, pillar structures, 15 μm in diameter with a height of 30 μm and spacing of 15 μm, were fabricated by a SU-8 negative photoresist (MicroChem Corp., Newton, MA, USA) on a 2″ mirror-polished silicon wafer (Ferrotec Silicon, Tokyo, Japan) according to the manufacturer’s protocol. The scanning electron microscope (SEM) image in Figure 2A was obtained using the 3D Real-surface view (VE-8800; KEYENCE, Osaka, Japan). PDMS prepolymer (Silpot 184; Corning Toray Co., Tokyo, Japan) mixed with a curing reagent was poured onto the mold, degassed, and cured at ~70°C for ~2 h. Then, the cured PDMS slab was peeled from the mold (Figure 2B). Within a single microchamber device, with a dimension of 2 cm ×2 cm, 2,160 chambers were arrayed, consisting of 4 subarrays of 9 ×60 chambers each. Each subarray was numbered for ease of spotting specific locations. To retain the buffer and drug solutions, a ring structure made of PDMS with an inner diameter of 10 mm was placed on the microchamber device (Figure 2C).

The surface of the microchamber device was coated with an extracellular matrix to enhance cell adhesion. Specifically, the device was incubated with a solution of 50 μg/ml human fibronectin (BD Biosciences, San Jose, CA, USA) for 30 min at room temperature. After washing with distilled–deionized water (DDW), the device was stored at 4°C. Prior to use, the chamber device was degassed for 10 min in a vacuum desiccator to avoid bubbles becoming trapped in the microchambers.

### Cell staining

To confirm cell adherence, imaging of cells with stained cellular membranes and nuclei was performed. HeLa cells attached to the microchamber device were incubated with 0.5 μg/ml Hoechst33342 (Life Technologies) and 10 μg/ml 1,1′-dioctadecyl-3,3,3′,3′-tetramethylindocarbocyanine perchlorate (DiI; Life Technologies) at 37°C for 20 min to stain the cell nucleus and cell membrane, respectively. After washing with BSS, cells were observed for fluorescence using a confocal laser scanning microscope.

### Microscopic observation

Cells with GFP and fluorescent markers that had adhered to the microchamber device were imaged using an inverted epi-fluorescence microscope (IX 51; Olympus, Tokyo, Japan) or a confocal laser scanning microscope (LSM-70; Carl Zeiss, Jena, Germany). Acquired fluorescent images were subjected to fluorescence intensity analysis using the ImageJ software. Statistical analyses were performed with GraphPad Prism software (GraphPad, San Diego, CA, USA) using the Student’s *t*-test.

### Experimental design and procedure

**Figure 3:**
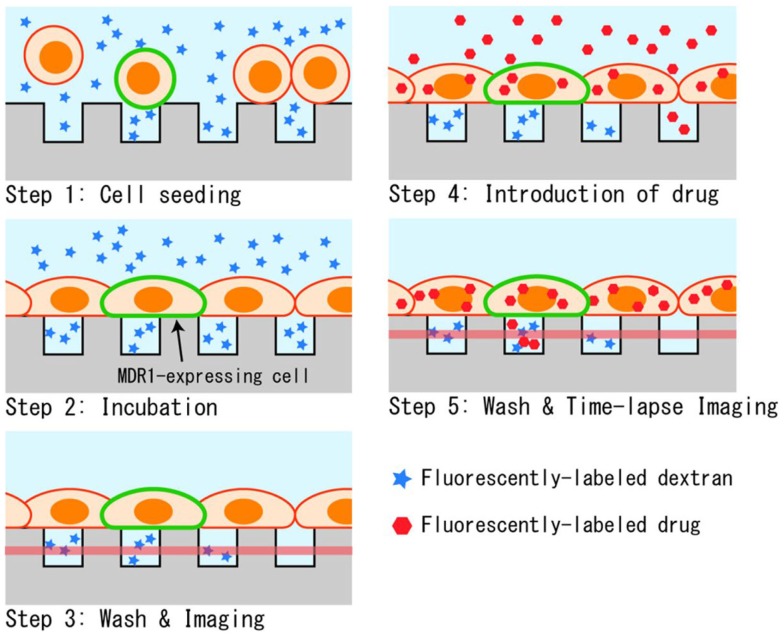
**Experimental procedure**. (1) Suspended cells in dextran solution are seeded on the microchamber device. (2) Cells adhere to form a confluent layer over the microchambers. (3) Buffer above the cells is washed and the sealing of chambers was confirmed by fluorescent imaging. (4) Drug is introduced, and incorporated into cells during incubation. (5) After the second washing, the drug transported into microchambers is measured by fluorescent imaging.

The measurement procedure for the drug transport assay in the designed microchamber device is depicted in Figure 3. First, cells that were detached with trypsin were seeded onto the microchamber. Cells precipitated on the surface of the microchamber device, and stayed at the surface instead of sinking into chambers since their diameters were smaller than the typical diameter of a cell (Figure 3, Step 1). The buffer used was supplemented with fluorescently labeled dextran. When the chamber device was incubated at 37°C for 90 min, cells spread to adhere and cover the chamber (Figure 3, Step 2). After washing with buffer, microchambers with confined spaces created by the cells were screened by observing the fluorescent dextran remaining in the chamber (Figure 3, Step 3). Following this, the buffer was exchanged for a buffer containing a fluorescently labeled anti-cancer drug, and the whole device was again incubated for 10 min (Figure 3, Step 4). At this step, the anti-cancer drug was incorporated and accumulated in the cells. Finally, the device was washed with the buffer to remove excess of the anti-cancer drug, and fluorescent imaging was performed (Figure 3, Step 5). When fluorescently labeled drugs were exported from the cells expressing transporters, as confirmed by the fluorescence of GFP, they accumulated within the confined spaces and fluorescence intensity increased in a time-dependent manner.

In the present system, three types of fluorescent substances were used: GFP as an expression marker of MDR1, fluorescent dextran as a marker to confirm the hermeticity of the chamber, and fluorescently labeled anti-cancer drugs. For imaging GFP, blue excitation was used, whereas for the anti-cancer drug, paclitaxel conjugated with BODIPY 564/570 (0.5 μM in concentration; Life Technologies), green excitation was used. To distinguish from these two fluorescent substances, cascade blue-conjugated fluorescent dextran (MW 3,000 or 10,000, 4 μM in concentration; Life Technologies), which can be observed under UV excitation, was chosen. With this strategy, analysis of transport activity was conducted on chambers covered by GFP-expressing cells and filled with dextran.

To confirm if the measured increase in fluorescence was the result of transporter activity and not from passive diffusion or other unknown reasons, we conducted the experiment with an inhibitor. In this case, 100 μM of verapamil (Sigma-Aldrich), an inhibitor of the MDR1 transporter, was added to all buffer solutions used in the assay, i.e., the cell suspension in Step 1, buffer with paclitaxel introduced in Step 4, and BSS used in Steps 3 and 5.

## Results

### Cell morphology in the microchamber device

**Figure 4:**
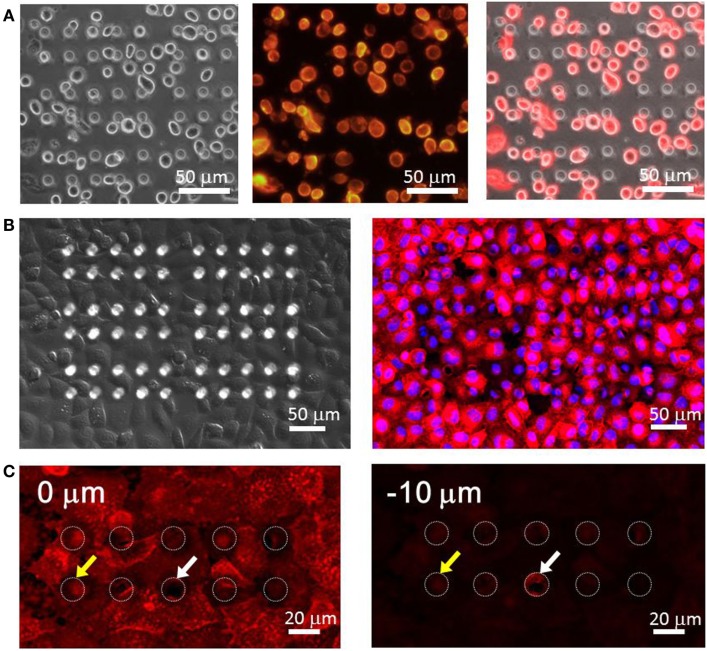
**(A)** HeLa cells right seeding on the microchamber device. (Left) differential interference contrast (DIC) image, (center) fluorescence image of the plasma membrane, (right) overlaid image. **(B)** HeLa cells spread over the microchamber device after incubation. (Left) DIC image, (right) fluorescence image. The red color shows the plasma membrane stained by DiI and the blue color shows the nucleus stained by Hoechst33342. **(C)** Imaging of the plasma membrane obtained at the surface of the chamber device (0 μm, left) and at the middle of the chamber (−10 μm, right).

We observed the adherence state and morphology of HeLa cells seeded in the microchamber device. From a 3.0 × 10^7^ cell/ml suspension of cells that were detached from the culture dish with trypsin, 500 μl was taken and used to seed cells in the device. Suspended cells were spherical with a diameter of 15–20 μm. Thus, when they precipitated and reached the upper surface of the chamber device, they did not fall inside the microchambers with openings of 15 μm in diameter (Figure 4A). The differential interference images taken after 90 min of incubation are shown in the left image of Figure 4B. Cells are shown to have spread and adhered over the device with 100% confluency. The morphology of cells adhered to the flat PDMS surface was identical to that on the plastic dish. As the morphology of cells overlapping with the chamber was difficult to observe under bright-field imaging, we conducted fluorescence imaging by staining cells. The overlaid image of nuclei stained with Hoechst33342 and cell membranes stained with DiI is shown in the right image of Figure 4B. The presence of hollows had minimal effect on the morphologies of both membranes and nuclei, and cells spread almost uniformly over the upper surface of the PDMS device. To further examine whether cells had penetrated into the microchambers, we compared microscopic images focusing on either DiI-stained cells on the device surface or the interior of chambers (10 μm below the cell surface) (Figure 4C). No membrane fluorescence was observed within most of the chambers, suggesting these microchamber spaces remained vacant. Further, no penetration was observed when the microchambers were completely covered with single cells. In some chambers, membrane fluorescence was observed, and in such cases, the edge of a cell overlapped with the opening of the microchamber. It is likely that the periphery of cells penetrated into the microchamber along the sidewall. Based on these results, HeLa cells seeded on this microchamber device were confirmed to spread consistently, to adhere, and to cover the microchamber.

### Confirmation of the sealing performance of the microchamber covered by cells

**Figure 5:**
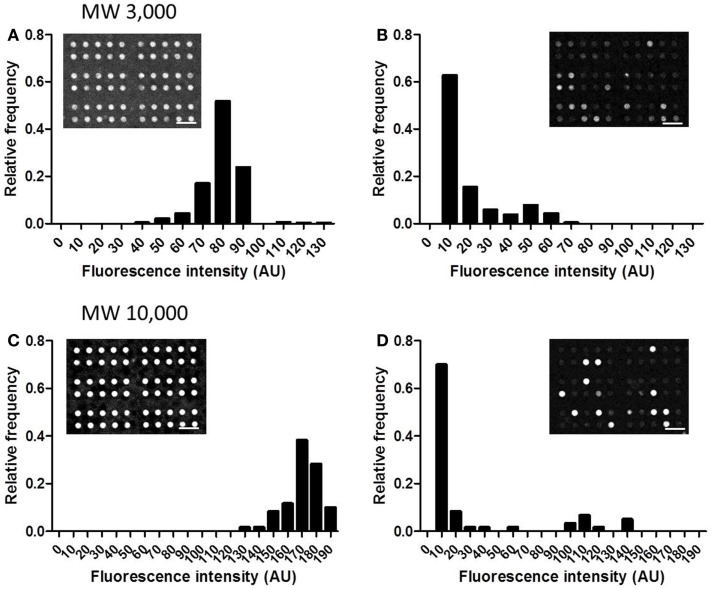
**Distribution of the intensity from fluorescent dextran retained in microchambers**. **(A)** MW 3,000, before washing. **(B)** MW 3,000, after washing. **(C)** MW 10,000, before washing. **(D)** MW 10,000, after washing. All scale bars in micrographs represent 50 μm.

In this device, small, confined spaces are created by attaching adherent cells over the openings of the microchambers, and exported drugs accumulate within these spaces. Thus, good sealing is required, so that drugs accumulated within the microchambers do not leak out. The sealing performance was tested using two types of fluorescent dextrans with MW of 3,000 and 10,000. After detaching cells from a culture dish, 4 μM of fluorescent dextran was added to the cell suspension. When cells reached confluency, the microchamber layer (i.e., the layer 10 μm below the cell layer) was observed before the washing step (Figure 3, Step 2). At this stage, fluorescence of both dextrans, with MW of 3,000 and 10,000, were uniform in all the microchambers (Figures 5A,C, inset). Later, when the buffer was exchanged at the washing step (Figure 3, Step 3), fluorescence disappeared in the majority of the microchambers, but residual fluorescence was still observed in some of the chambers (Figures 5B,D, inset). A histogram in Figure 5 demonstrates the distribution of fluorescent intensity derived from all 540 chambers in a single experiment. In the experiment with MW 3,000 dextran, two distinct peaks were clearly observed after washing (Figure 5B), indicating the microchambers at the higher intensity peak were closed. The fraction of microchambers with a fluorescent intensity of 40 or above, the value at the valley of the peaks was 18.2%. In the case of MW 10,000 dextran, a similar fraction of microchambers was closed. Microchambers with an intensity above 40 were 20.2% of the total number of chambers (Figure 5D). That is, ~20% of the chambers had achieved a sealing performance high enough so that the fluorescent dextran would not be washed away.

**Figure 6:**
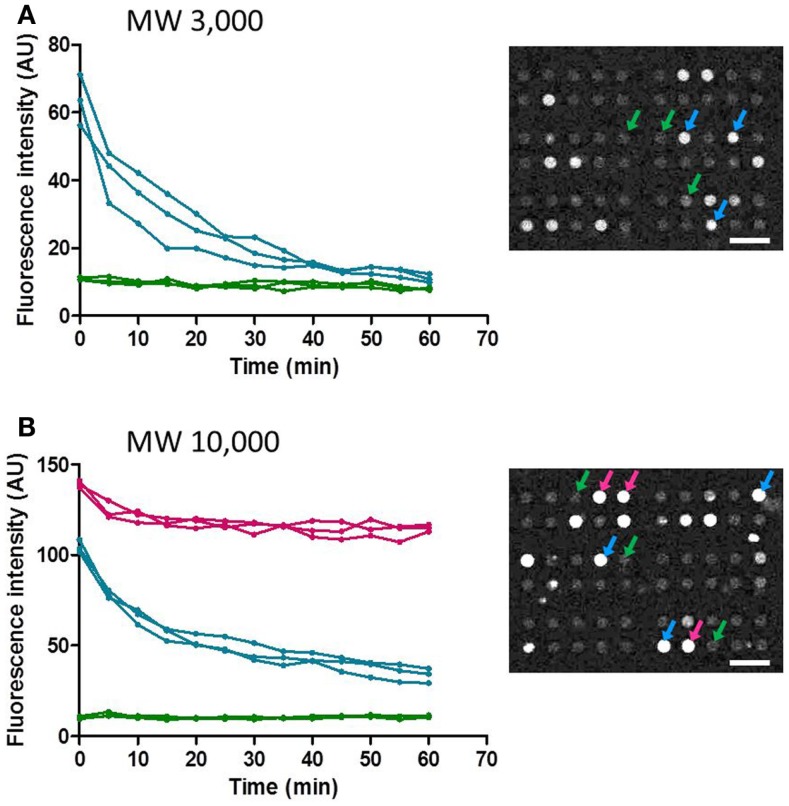
**Time-course of the fluorescence intensity retained in the selected microchamber**. Data points obtained at every 5 min were plotted for clarity. **(A)** MW 3,000. **(B)** MW 10,000. All scale bars in micrographs represent 50 μm.

Next, temporal changes in fluorescent intensity within the microchambers after washing were observed. Images were taken every 5 min, and time-course data for representative microchambers are shown in Figure 6. In the case of fluorescent dextran with a MW of 3,000, in most of the microchambers that retained fluorescent dextran after the buffer wash, the fluorescent intensity tended to decrease in a time-dependent manner to ~30% of the starting intensity within 40 min after the wash. In contrast, no time-dependent change was observed in microchambers in which no fluorescence was observed immediately after the buffer wash (Figure 6A). In the case of fluorescent dextran with a MW of 10,000, three patterns of changes in intra-chamber fluorescent intensity were observed (Figure 6B). In microchambers with high fluorescent intensity immediately after the wash, the decrease in fluorescent intensity was only about 10% at *t* = 60 min. In microchambers with intermediate fluorescent intensity immediately after the wash, the fluorescent intensity decreased to about 50% within 30 min of the wash, in comparison with the intensity observed immediately after washing. In microchambers with almost no fluorescence immediately after the wash, almost no time-dependent change was observed. The proportions of microchambers demonstrating each of these three patterns were 5, 15, and 80%, respectively.

### Transporter activity assay

**Figure 7:**
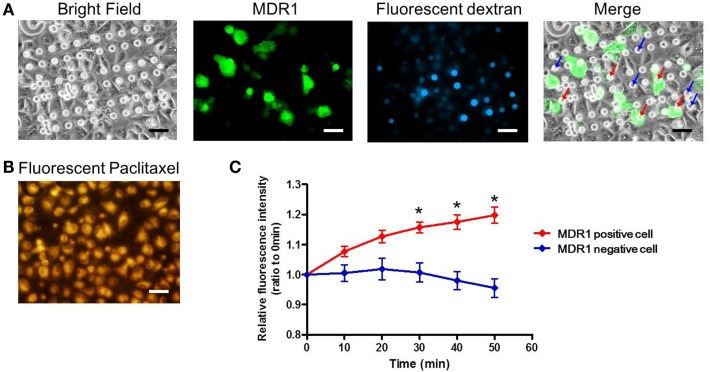
**Drug transport assay**. **(A)** Bright and fluorescence images, showing the cell morphology, MDR1 expressed in the cell, and fluorescent dextran retained in the microchamber, respectively. Microchambers enclosed by the MDR1-expressing cell can be spotted in the merged image. **(B)** Fluorescent image showing paclitaxel incorporated into the cell after incubation. **(C)** Time-course of fluorescence intensity in the microchambers covered by MDR1 positive cells (red line) and MDR1 negative cells (blue line), both retaining dextran. Each line represents the average of five independent chambers indicated by the arrows with corresponding colors in the merged image. Data are presented as the mean ± SEM. *P < 0.05 by Student’s *t*-test. All scale bars in micrographs represent 50 μm.

**Figure 8:**
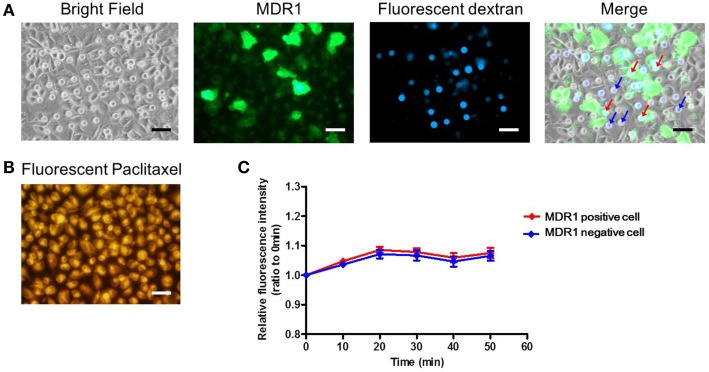
**Drug transport assay with the inhibitor**. **(A)** Bright and fluorescence images, showing the cell morphology, MDR1 expressed in the cell, and fluorescent dextran retained in the microchamber, respectively. Microchambers enclosed by the MDR1-expressing cell can be spotted in the merged image. **(B)** Fluorescent image showing paclitaxel incorporated into the cell after incubation. **(C)** Time-course of fluorescence intensity in the microchamber for MDR1 positive (red line) cells and MDR1 negative cells (blue line) both retaining dextran. Each line represents the average of four independent chambers indicated by the arrows with corresponding colors in the merged image. Data are presented as the mean ± SEM and significance was assessed using the Student’s *t*-test. All scale bars in micrographs represent 50 μm.

Finally, we conducted the transporter activity assay with HeLa cells over-expressing the ABC transporter MDR1. After achieving confluency on the microchamber device, a buffer containing fluorescent dextran was exchanged with BSS. At this stage, images of fluorescent dextran and GFP were obtained. From these images, we located the microchambers where cells expressing GFP fused to MDR1 were covering chambers to form a confined space for later analysis (Figure 7A). Next, the buffer with 0.5 μM paclitaxel, an anti-cancer drug, conjugated with BODIPY 564/570 was introduced. After incubation for 10 min the excess drug was washed out. The cellular uptake of fluorescent paclitaxel, due to passive transport across the plasma membrane, was confirmed by the observation of intense red fluorescence in cells (Figure 7B). Following this, time-lapsed images were obtained. Since paclitaxel is known to bind to tubulin (Jordan et al., 1996; Jordan and Wilson, 1998) and microtubules, fluorescent paclitaxel remained in cells without MDR1 for more than an hour, which was consistent with previous literature (Jang et al., 2001). However, in the microchambers covered by MDR1-expressing cells and with enclosed fluorescent dextran, a time-dependent increase in fluorescence intensity was observed (Figure 7C red line). Conversely, in microchambers covered by cells without MDR1 but with fluorescent dextran enclosed, no increase in fluorescence intensity was observed (Figure 7C blue line). Note that statistically significant differences were observed at 30, 40, and 50 min after starting the measurement. We also conducted the experiment in the presence of the MDR1 inhibitor, verapamil. In this case, no obvious differences were observed in the cell morphology and expression of MDR1 (Figures 8A,B). However, no significant differences were observed within microchambers with cells either expressing or not expressing MDR1 and with dextran enclosed in both (Figure 8C). The fluorescence intensity with paclitaxel in every chamber remained unchanged.

Based on these results, the transporter-dependent accumulation of paclitaxel within the confined spaces of microchambers was confirmed. Furthermore, it is worth mentioning that, as the size of chamber openings was smaller than the size of cells, the observed concentration increase represents the increase from a single cell.

## Discussion

In this study, we aimed to develop a microchamber device for directly detecting drug transport via transporters of adherent cells. We examined the extent of the formation of confined spaces by coverage with adherent cells seeded on the microchambers. Furthermore, by using cells over-expressing the transporter protein, we successfully detected the transport activity of fluorescently labeled anti-cancer drugs at the single-cell level.

We used HeLa cells as our model cell line and found that these cells spread to confluence over the microchambers with diameters of 15 μm, without falling or penetrating into the microchambers. As a result, ~20% of the chambers were in a closed state so that the contents would not be washed away when rinsed with a buffer. However, the sealing performance was not high enough to retain molecules with a MW of 3,000 (~1.6 nm in gyration radius) (Fishman et al., 1987). For molecules with a MW of 10,000 (~2.9 nm in gyration radius), complete sealing was achieved in about 5% of the chambers within the assay period. In general, cells attached to the extracellular matrix at points called focal adhesions (FAs) (Geiger et al., 2001; Berrier and Yamada, 2007), so that the presence of shallow spaces in between was expected. As HeLa cells do not form tight junctions, which are seen between epithelial cells, it is likely that they cannot achieve the sealing performance to completely prevent nanometric molecules from diffusing out. The ability to form a hermetic seal is expected to depend on the cell type and size of chamber openings, both areas that we will continue to investigate.

The MW of the substrate (fluorescent paclitaxel) used in this study for the transport assay of an anti-cancer drug was 1,099. Therefore, in this work, the drug transported into a microchamber should not be completely retained and likely diffused out at a certain rate. Nevertheless, a significant increase in fluorescent intensity was observed in association with paclitaxel transport into the chambers enclosed by MDR1-expressing cells. This was likely due to the accumulation of the drug when transport exceeded leakage by passive diffusion through the gap between cells and the device wall, although further investigation is needed to verify this hypothesis.

This work provides the first step toward the rapid, easy detection of transporter activity at the single-cell level, by directly using intact adherent cells to form closed microchambers. The fact that the accumulation of fluorescent substrate transported into a microchamber was measurable using a standard cell line (e.g., HeLa cell) suggests that our strategy is likely to be applicable to other adherent cells, including cells from clinical samples. The device used in this study was different from the transwell device described in the introduction. Our device does not require complete confluence of cells and can be used even in a case where the distribution of cells is patchy. Therefore, an assay can be conducted with trace amounts of samples. Furthermore, as the detected transporter activity is intrinsically from a single cell, this assay may be applicable to investigating cellular diversity in cancer tissues or to detecting rare cells in a population in future studies. To facilitate these applications, the sealing property of microchambers should be well characterized for various cell types, culture conditions, and sizes of microchamber openings. We will continue investigating this issue to increase the scope of applications for this device.

## Author Contributions

MT conceived and designed the experiments, performed the experiments, analyzed the data, and wrote the manuscript. EU conceived and designed the experiments, and provided conceptual advice. HS conceived and designed the experiments, performed the experiments, and wrote the manuscript.

## Conflict of Interest Statement

The authors declare that the research was conducted in the absence of any commercial or financial relationships that could be construed as a potential conflict of interest.
